# Dr. Bhau Daji Lad (1822–1874): A Pioneer in Integrative Medicine and Social Reforms for Leprosy

**DOI:** 10.7759/cureus.78684

**Published:** 2025-02-07

**Authors:** Anish A Deshmukh, Sonali G Choudhari

**Affiliations:** 1 Computer Science, Manipal University, Jaipur, IND; 2 Community Medicine, Jawaharlal Nehru Medical College, School of Epidemiology and Public Health, Datta Meghe Institute of Higher Education and Research, Wardha, IND

**Keywords:** historical vignette, indigenous medicine, integrative medicine, leprosy treatment, public health, social reformer

## Abstract

Dr. Bhau Daji Lad (1822-1874) was an eminent Indian physician and scholar who made significant contributions to medicine, education, and social reform. Hailing from Goa, he graduated from Grant Medical College, where he is regarded as the father of its foundation. His pioneering work in integrating Western medicine with traditional Indian practices became particularly notable when he successfully treated leprosy using chaulmoogra oil. Dr. Lad was also a prominent advocate for public health, hygiene, and educational reforms. His legacy is honored at the "Dr. Bhau Daji Lad Museum" in Mumbai, which showcases his valuable contributions to the cultural and intellectual development of the city.

## Introduction and background

**Figure 1:**
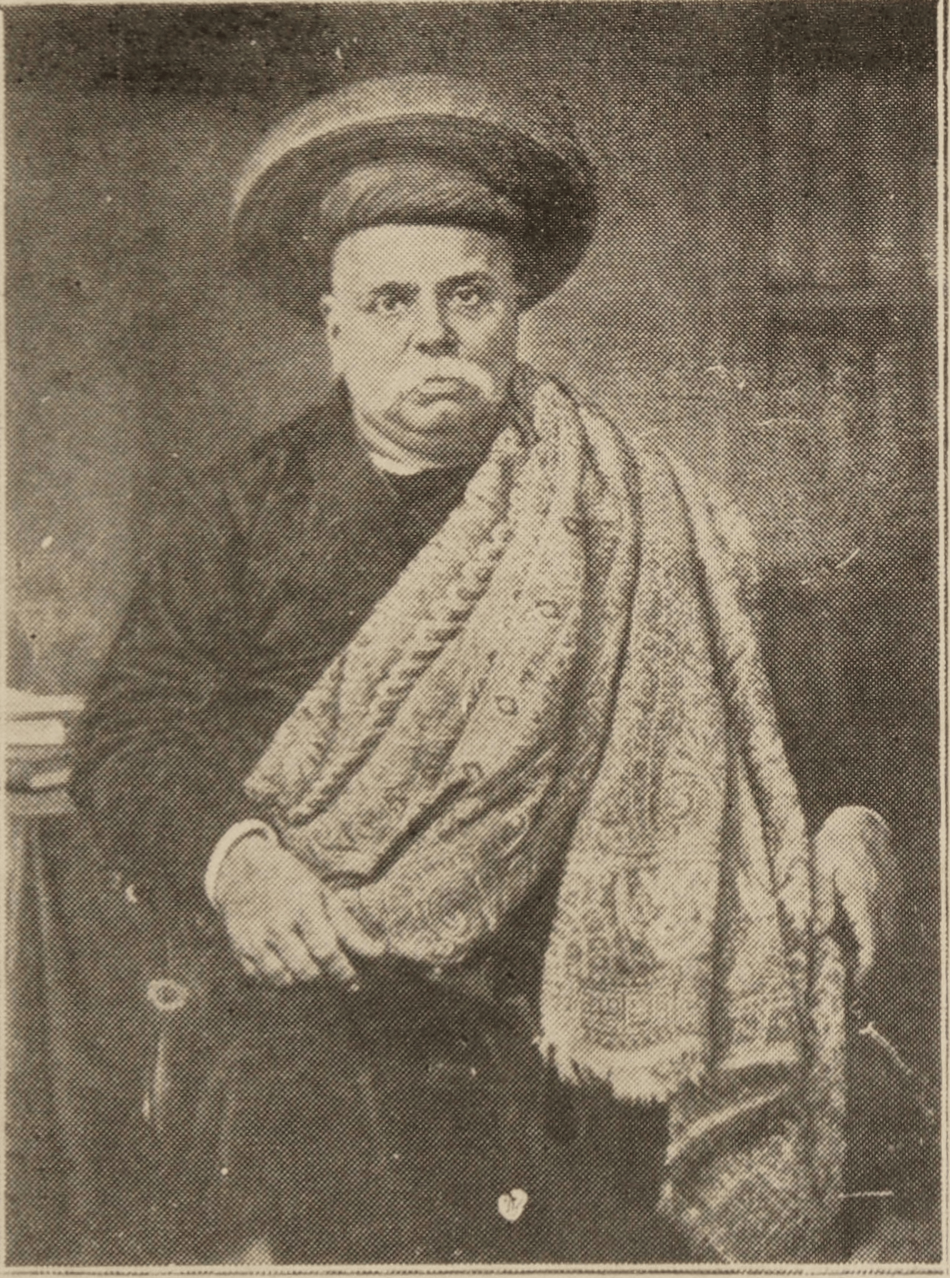
Dr. Bhau Daji Lad Source: [1] Credit: This file is in the public domain.

Dr. Bhau Daji Lad (Figure 1) [1] was a renowned Indian physician, Sanskrit scholar, and antiquarian who made significant contributions to medicine. He was a pioneer in integrating Western medicine with traditional Indian medicine and is also known as the man responsible for introducing many new treatments and research undertakings [2]. Dr. Lad played a pivotal role in the political and social history of Bombay (now Mumbai), India. In addition to his medical achievements, he was a distinguished historian, amateur botanist, advocate for girls' education, and a passionate supporter of the arts [3]. Despite his humble beginnings, his exceptional intellect and unwavering determination propelled him to great heights [4].

## Review

Early life

Bhau Daji was born in 1822 to a poor Saraswat Brahmin family in the village of Manjare, within the native state of Sawantwari, Goa. His birth name was Ramkrishna Lad. His father, Vithal, affectionately called him “Bhau” [2,4]. A child prodigy, Bhau Daji's exceptional intelligence became evident early on. His remarkable talent in chess caught the attention of the Governor of Bombay, the Earl of Clare, who encouraged his father to pursue a Western education for him [2,5].

Education

Bhau enrolled at the prestigious Elphinstone College in Bombay in 1840. By 1843, he had taken on the role of Assistant Teacher of Chemistry and Natural Philosophy at the same institution [2,6]. In 1844, the British Government organized an essay competition on female infanticide in Kutch and Kathiawar [7], in which Bhau won first prize. His academic prowess continued to shine, leading him to join the inaugural batch of Grant Medical College, where he completed his medical degree, setting the stage for his distinguished career [8].

State of medicine in India during the 19th century

Between 1757 and 1900, the popularity of Western medicine in India steadily increased, which was indeed largely encouraged and supported by the English and Indians who were educated in the West. However, only a few qualified Indians became practitioners of Western medicine, with the majority of doctors being Europeans or subordinate medical personnel. Over time, the colonial government reduced funding for indigenous medical practitioners, diminishing the status of vaidyas and hakims, who were often considered less rational than their Western practitioners. While some traditional practitioners defended their systems, others incorporated Western methods into their practice. Meanwhile, European doctors focused primarily on researching medicinal plants and tropical diseases, with limited engagement with Indian medicine practitioners [9]. Indian medical traditions, including Ayurveda, are marked by diversity and shaped by interactions with colonialism and nationalism. Within the colonial context, Ayurveda was often viewed as a "subaltern" science compared to Western medicine. After the 1820s, initial interest in Ayurveda shifted toward an antiquarian fascination with inexpensive herbal remedies, largely overlooking its theoretical underpinnings [10]. 

Dr. Lad's contributions to medicine

Establishment of Grant Medical College

Grant Medical College, founded in 1843, was one of the first European medical schools in India, designed to train Indian practitioners. Dr. Bhau Daji Lad was among its first students, graduating in 1851. Following his graduation, he opened a dispensary with his brother and became a strong advocate for establishing a medical institute that would teach Western medicine to Indian students. This period marked a pivotal moment in the evolution of Indian medical education [6-8].

Integration of Western and Indian Medicine

Dr. Lad was a visionary who sought to create a healthcare system that combined the effectiveness of modern medicine with traditional practices. He believed that integrating the best aspects of Ayurveda with modern medical technologies would significantly enhance the healthcare system. As a student of both Eastern and Western education, Dr. Lad had a unique ability to merge these two knowledge systems. His deep understanding of Sanskrit texts complemented his knowledge of Ayurveda, which had a rich repository of therapeutic practices for diseases that had long plagued the Indian subcontinent. Throughout the 1860s, Dr. Lad conducted rigorous research, adopting a Western experimental approach to systematically investigate the remedies used by indigenous communities. He devoted considerable time to studying Sanskrit medical treatises and the properties of native herbs, experimenting with how traditional cures could be incorporated into his practice to improve patient care [11].

Treatment of Leprosy

Dr. Lad’s groundbreaking work in diagnosing and treating leprosy is a central part of his medical legacy. He meticulously explored the various aspects of the disease and remained relentless in his search for effective treatment options. His pioneering use of Chaulmoogra oil, derived from the seeds of *Hydnocarpus wightiana*, became a key breakthrough. Through careful observation and trials, Dr. Lad became the first to employ this oil in leprosy treatment, offering hope to countless individuals afflicted by the disease. His approach exemplified his belief in integrating ancient Indian medical knowledge with modern Western practices, positioning him as a pioneer in the field of integrative medicine, a discipline that is now gaining prominence, especially in the United States. The treatment he developed, which he named the "Bhau Daji" method, was widely discussed and adopted [11-13].

Unfortunately, Dr. Lad’s method for treating leprosy sparked debate among healthcare professionals of the time, with some expressing skepticism. Although Dr. Lad intended to publish his findings, his sudden illness and death prevented him from doing so. While the effectiveness of his treatment remains debated, his work brought significant attention to Chaulmoogra oil, which remained the primary treatment for leprosy until the introduction of dapsone in 1941. Despite its importance, Bhau Daji Lad’s contributions have often been overlooked in medical literature [12].

Literature on Chaulmoogra Oil's Efficacy 

Chaulmoogra oil, a fixed oil obtained from the ripe seeds of *Hydnocarpus *species, has long been used in traditional medicine for treating leprosy and other skin diseases. Pharmacological studies have shown that the oil exhibits potential activity against *Mycobacterium leprae* and is particularly effective in treating early-stage leprosy cases [14]. Native to Thailand, Myanmar, and India, Chaulmoogra oil contains unsaturated fatty acids that give it a strong bactericidal effect against the bacteria responsible for leprosy and tuberculosis. For centuries, it has been used in Ayurveda to treat a variety of conditions, including leprosy, tuberculosis, gout, and skin disorders [15].

Public Health and Hygiene Advocacy

Dr. Lad was a strong advocate for public health and hygiene, recognizing the critical role of disease prevention in improving overall health. He actively supported sanitation initiatives and raised awareness about cleanliness among the general public. Dr. Lad led sanitation and hygiene campaigns, addressing public health crises such as cholera and plague outbreaks. His advocacy for better sanitation infrastructure played a pivotal role in improving public health outcomes, particularly in urban areas [2,5].

Reforms in Healthcare Systems

A vocal critic of the existing healthcare system in India, Dr. Lad called for comprehensive reforms, focusing on accessibility, quality of care, and healthcare education. His efforts contributed to positive changes within the healthcare system. He was involved in medical associations that aimed to elevate professional standards and accountability, pushing for the establishment of more healthcare facilities in underserved areas to ensure marginalized communities had access to essential medical services.

Preservation of Medical Heritage

Dr. Lad was also a passionate collector of ancient medical manuscripts and artifacts, recognizing the importance of preserving India's rich medical heritage. To house his collection, he established a museum that today bears his name-the "Dr. Bhau Daji Lad Museum." This museum stands as a testament to his commitment to safeguarding the history of medicine and promoting knowledge in the field [16]. 

Dr. Bhau Daji Lad Museum

The Lancet, an English daily, once wrote on January 13, 1855: “To (Dr Bhau Daji’s) exertions Bombay will owe the Economic Museum and Zoological Gardens, and the various galleries of science and art now in process of the organisation.” The Dr. Bhau Daji Lad Museum, the oldest museum in Mumbai, first opened to the public in 1857. It was the first colonial building specifically constructed to house a museum. Today, the museum stands as one of the city's most significant historical landmarks [17]. 

The museum became a valuable repository of information on various communities and Mumbai's industrial arts. The construction of the museum building took a decade, primarily due to insufficient funding. In 1858, a committee was formed, consisting of Jugonnath Sunkarset, George Birdwood, Dr. Bhau Daji Lad, and other patrons, to lead fundraising efforts for the building’s establishment and to expand the museum's collection with the finest specimens of "Indian manufactures." Dr. Lad played a pivotal role in persuading citizens to contribute generously to the museum. The government also provided financial support [18]. 

Over a century later, on November 1, 1975, to commemorate the 100th anniversary of his death, the museum was renamed the "Dr. Bhau Daji Lad Museum" (formerly the Victoria and Albert Museum) in recognition of Dr. Lad's significant role in the cultural development of Mumbai and his vision, which led to the museum's establishment. Dr. Lad, who was the first Indian Sheriff of Mumbai, was also a philanthropist, historian, physician, surgeon, and the secretary of the museum committee when it was first founded [16,19]. The museum stands as a testament to his multifaceted contributions and serves as a repository of India’s rich cultural heritage.

Lad's contributions beyond medicine

Dr. Bhau Daji Lad contributed far more than just his medical expertise. A true polymath, he had a deep interest in archaeology, history, and social welfare, approaching each with equal passion. He became an expert epigraphist and numismatist, further cultivating his love for India’s rich heritage [2,5,12].

As a social reformer, Dr. Lad advocated for women's rights and strongly condemned social vices such as infanticide. He championed women’s education and worked to uplift the poor by defending their rights. He also played a leading role in establishing academic institutions that influenced the intellectual climate of Bombay. On occasion, he expressed a keen interest in the commercial and industrial development of the country [11,12,18].

In addition to his accomplishments in medicine and the arts, Dr. Lad was a prolific writer. His notable works include a descriptive catalog of the Government Central Museum, contributions to Marathi literature, and essays on various subjects such as history, archaeology, and cultural heritage [20]. A respected Sanskrit scholar, he published several articles in the Royal Asiatic Society Journal and was renowned for his expertise in interpreting the ancient Elephanta inscriptions [21].

Legacy

Dr. Bhau Daji Lad’s legacy extends beyond his medical achievements. He was a visionary leader who championed the advancement of medical education, the integration of Western and Indian medicine, and the improvement of public health and sanitation. His contributions have had a lasting impact on the country’s medical development [5,12].

Tragically, Dr. Lad passed away in 1874 at the age of 52. Despite his short life, he left an extraordinary legacy. His pioneering spirit, intellectual brilliance, and unwavering service to humanity continue to inspire generations [22].

## Conclusions

Dr. Bhau Daji Lad was Bombay’s first medical graduate from Grant Medical College and emerged as a towering figure in Indian medicine and intellectual history. By seamlessly integrating Ayurveda with Western medicine, Dr. Lad laid the foundation for a holistic approach to healthcare in India, a vision that remains profoundly relevant today. He was a man of versatile achievements: a renowned educationist, politician, and social reformer. His life story serves as an inspiration to generations of doctors and scientists, encouraging them to embrace the synergy between traditional and modern approaches to medicine. Dr. Lad actively participated in research across various fields, including museums and theater, and was a staunch advocate for the Indian voice through the Bombay Association. His legacy continues to resonate in the city’s major institutions. His unwavering commitment to the upliftment of Indian society earned him widespread respect and admiration.
